# Translation and cross-cultural adaptation of the Mayo High Performance Team Scale (MHPTS) into Brazilian Portuguese

**DOI:** 10.1590/0100-6991e-20243740-en

**Published:** 2024-07-02

**Authors:** MARCOS MACIEL CANDIDO JUSTINO DOS SANTOS, SARA FITERMAN LIMA, ALEXANDRE SLULLITEL, NELSON GONÇALVES DE OLIVEIRA, ELAINE CRISTINA NEGRI SANTOS, EDUARDO CRUZ ARAÚJO, DANIELLE BRUGINSKI, GERSON ALVES PEREIRA

**Affiliations:** 1 - Universidade de São Paulo, Programa de Pós-Graduação em Ciências da Reabilitação HRAC - Bauru - SP - Brasil; 2 - Universidade Federal do Maranhão, Programa de Pós-Graduação em Saúde Coletiva - São Luís - MA - Brasil; 3 - Universidade de São Paulo, Programa de Pós-Graduação ACCEPT - São Paulo - SP - Brasil; 4 - Universidade do Oeste Paulista - UNOESTE, Curso de Medicina - Preaidente Prudente - SP - Brasil; 5 - Carefy - Ribeirão Preto - SP - Brasil; 6 - Faculdades Pequeno Príncipe - Curitiba - PR - Brasil

**Keywords:** Simulation Training, Interprofessional Education, Health Human Resource Training, Treinamento por Simulação, Educação Interprofissional, Capacitação de Recursos Humanos em Saúde

## Abstract

**Objective:**

describe the process of translation and cross-cultural adaptation of the Mayo High Performance Team Scale into Brazilian Portuguese.

**Method:**

descriptive study of validation and cross-cultural adaptation of the scale, carried out virtually, following assumptions proposed by Beaton and collaborators. It had a sample of 40 experts, and carried out two rounds, one for validation and one for final assessment.

**Results:**

after following all translation steps, the scale was presented to the committee of experts who reached a consensus (IVC between 0.9 and 1.0) that there was no discrepancy, after evaluating the semantic, idiomatic, experiential and conceptual equivalences between the original scale and the translated version.

**Conclusion:**

The Brazilian Portuguese version of the MHPTS was adequately translated and validated, revealing excellent potential for use in clinical simulation contexts for multidisciplinary scenarios.

## RESUMO

**Objetivo:**

descrever o processo de tradução e adaptação transcultural da escala Mayo High Performance Team Scale para o português brasileiro.

**Método:**

estudo descritivo de validação e adaptação transcultural da escala, realizado de maneira virtual, seguindo pressupostos propostos por Beaton e colaboradores. Contou com uma amostra de 40 especialistas, e realizou duas rodadas, sendo uma de validação e uma de apreciação final.

**Resultados:**

após seguir todas as etapas de tradução a escala foi apresentada ao comitê de especialistas que chegou a um consenso (IVC entre 0,9 e 1,0) sobre não haver qualquer discrepância, após avaliação das equivalências semântica, idiomática, experiencial e conceitual entre a escala original e a versão traduzida.

**Conclusão:**

A versão em português brasileiro da MHPTS foi adequadamente traduzida e validada, revelando excelente potencial de utilização em contextos de simulação clínica para cenários multiprofissionais.

## INTRODUCTION

Health care errors, once considered rare, are now a global concern^1^. The report “To err is human: Building a safer health system”, published to introduce the concept of a safer environment for health care, showed that such errors are much more frequent than imagined. In addition, it concluded that such errors are not the result of isolated individual actions, but of failures in systems, processes, and several other inadequate conditions^1^ ^-^ ^3^.

Among the latent conditions of risk for patient safety are protocols, work environments, communication, and teamwork, and it is estimated that 70-80% of health care errors can be attributed to failures in non-technical skills from health professionals^2^ ^,^ ^4^ ^-^ ^6^.

Although the interference of human factors for the performance of technical tasks has been discussed for a long time, it was in aviation that the non-technical skills training system was initially adopted, after a series of air disasters in the 1970s. This training, initially known as Cockpit Resource Management, was later expanded and adapted to other professional environments, such as companies and health services, and came to be called Crew Resource Management, Crisis Resource Management (CRM), or even Corporate Resource Management^7^ ^-^ ^10^. 

It is worth noting that, regardless of the terminology used, the acronym CRM has become synonymous with a quick and effective response to risk situations involving action under stress and time pressure for action^10^. Therefore, CRM can be defined as a management system with optimized use of available resources, such as human resources, operational procedures, and equipment, aiming to promote operational safety in critical situations^11^.

The goal of CRM training is to improve staff performance and minimize the risk of errors in performing a complex task. The method includes the use of techniques for team training, focusing on systems and cultures, rather than individuals or failures, where simulation is the teaching method usually used to train their principles of non-technical skills, task management, teamwork, situational awareness, and decision-making, can be practiced safely^12^. In addition, in simulation it is possible to identify how the multiprofessional team is performing these skills^2^ ^,^ ^13^. 

To assess teamwork and CRM skills in simulation-based education environments, a widely used instrument is the Mayo High Performance Team Scale (MHPTS), developed by Malec et al.^14^.

The MHPTS is comprised of eleven key determinants of effective crisis management, including effective communication, situational awareness, anticipating and planning events, designating the leader, establishing roles, distributing tasks, setting priorities, adapting to change, monitoring performance, and debriefing^15^.

Simulation training programs should be evaluated using valid and reliable instruments^16^. In this context, to contribute to the evaluation of teamwork in simulated scenarios, the objective of this article is to describe the process of translation and cross-cultural adaptation of the MHPTS scale to the Brazilian Portuguese.

Although there are other scales available to assess psychometric skills in teamwork, we chosen the MHPTS because it is a scale that has already been translated into other languages and is recognized for use in health care^15^ ^,^ ^16^.

## METHODS

**Figure 1:**
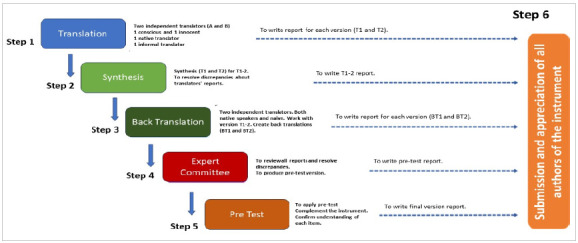
Guideline for conducting translation and cross-cultural adaptation of instruments.

This is a descriptive study of cross-cultural adaptation and validation of the scale for the assessment of non-technical skills of a multidisciplinary team, developed by the Mayo Clinic^14^, using as a reference the assumptions of the method of Beaton et al.^1^, which indicate a process composed of six stages (Figure 1).

The translation process aimed at equivalence between the original scale and the version adapted to a culturally diverse environment, since the target population of the scale speaks another language and lives in another country^17^. 

We used the guidelines for cross-cultural adaptation of instruments proposed by Beaton et al. as reference^17^, which indicate a process consisting of six steps (Figure 1). 

The composition of an Expert Committee is essential to achieve cross-cultural equivalence of the translated instrument, and should include professionals with experience in methodology, health care, and languages, as well as all translators and the observer who participate in the translation synthesis^18^. 

To validate the translated version, we invited 62 experts, of whom 40 accepted and composed the study sample. The members of this committee had experience in methodology, research, simulation, statistics, or intensive care and were all bilingual (Portuguese and English), all of them being professionals with notorious knowledge in simulation applied to the health area. In addition, four translators and one observer participated in the evaluation, accompanying all the validation work by experts. 

The validation process with the expert committee took place in a virtual environment through Google Forms^®^, with those who answered the instrument completely and within the deadline established for this stage participating in the study. We analyzed the collected data with Microsoft Excel^®^ software, version 2019. 

**Figure 2:**
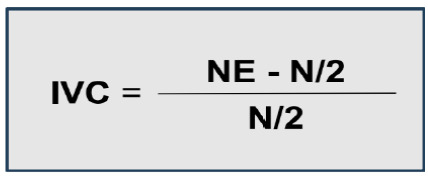
Calculation of the CVI based on the concordant answers given by the judges.

For the validation of the clinical scenario sections, we computed the Content Validity Index (CVI). The CVI is a measure of the experts’ agreement on the topics evaluated regarding the scenario elaborated and is calculated by adding the answers of the Likert scale (with answers 4 and 5 being considered as agreeing) and dividing them by the total number of answers (Figure 2). For this calculation, items that obtain 80% or more of agreement among experts can be considered validated^19^.

In this equation for calculating the CVI, we have NE, which refers to the number of specialists who agree with a parameter, and N, which is the total number of specialists participating in the research^20^.

The study complied with the recommendations of Resolution No. 466/2013 of the National Health Council, was submitted for consideration by the Ethics in Research Committee of the Universidade do Oeste Paulista, and approved by the Certificate of Presentation for Ethical Appraisal No. 63842122.0.0000.5515 and Opinion 5.743.901.

## RESULTS AND DISCUSSION

To ensure an accurate and reliable assessment of non-technical skills (NTSs) during team simulation training activities, valid and adequate instruments are needed. However, there are few instruments produced and/or validated for this purpose in the national scenario. Thus, the translation into Brazilian Portuguese was carried out, with cross-cultural validation of the MHPTS^14^ ^,^ ^16^.

The MHPTS evaluates NTSs, or soft skills, but it is worth noting that although this terminology is classically used and recognized, defining such skills is complex, starting with their nomenclature, which may have a connotation as not suitable to be trained and developed by repetition or to be of secondary importance to health professionals^20^ ^,^ ^21^. 

Thus, before starting, we secured the prior endorsement and authorization of the authors of the MHPTS, so that the original text could be translated and cross-culturally adapted to the Brazilian Portuguese, and from there continue its stages of translation and cross-cultural adaptation.

The first stage was the translation (forward) of the original instrument. This is an overly complex activity, because when translating an instrument, several types of equivalence in relation to the original must be sought, such as cultural, semantic, technical, content, criterion, and conceptual^17^.

At this stage, at least two direct translations of the instrument from the original (source) language into the target one are recommended. Bilingual translators, whose mother tongue is the target language, produce independent translations and written report with additional comments, highlighting challenging phrases or uncertainties, and justification for their choices. Thus, translations can be compared and discrepancies observed^17^.

Thus, this stage involved the presentation of the MHPTS to two independent translators (translators A and B), whose native language is Brazilian Portuguese. These translators produced two Portuguese versions, which were given the abbreviations T-1 and T-2. To meet the recommendations, the first translator had no knowledge of the concepts or any background associated with the area of application of the research (lay translator), while the other was aware of the concepts being evaluated. Both produced reports for this stage^17^ ^,^ ^18^.

The second stage, the synthesis of the translations, should take place with the two translators and an observer who will jointly synthesize the results of the translations obtained, discuss the divergences, and generate a version with the necessary adjustments^17^. Thus, based on the original MHPTS in English and the versions of the first (T1) and the second (T2) translators, a synthesis of the translations was carried out (producing a common translation T1-2), as well as a written report carefully documenting the synthesis process. (Table 1).

**Table 1:** T1 and T2 translation versions and version of choice.

ORIGINAL	T1	T2	T1-2
Mayo High Performance Teamwork Scale	Escala Mayo de trabalho em Equipe de Alto Desempenho	Escala Mayo de alto desempenho em equipe	Escala Mayo de trabalho em Equipe de Alto Desempenho
Use the following scale to rate the team on each dimension:	Use a seguinte escala para avaliar o time em cada dimensão:	Use a seguinte escala para classificar a equipe em cada aspecto:	Use a seguinte escala para avaliar o time em cada dimensão:
0 Never or Rarely	0 Nunca ou raramente	0 Nunca ou raramente	0 Nunca ou raramente
1 Inconsistently	1 Inconsistente	1 Inconsistente	1 Inconsistente
2 Consistently	2 Consistente	2 Consistente	2 Consistente
Please rate conservatively. Most teams that have not worked extensively together do not consistently demonstrate many of the qualities described in the scale.	Por favor, avalie cuidadosamente. Muitas equipes que não têm trabalhado bem juntas não demonstraram de forma consistente, muitas das qualidades descritas na escala.	Por favor, avalie de forma conservadora. A maioria das equipes que não trabalharam extensivamente juntas não demonstra consistentemente muitas das qualidades descritas na escala.	Por favor, avalie cuidadosamente. Muitas equipes que não trabalham adequadamente juntas não demonstram de forma consistente muitas das qualidades descritas na escala.
Always rate items 1-8.	Sempre avalie os itens 1-8.	Sempre avalie os itens de 1 a 8.	Sempre avalie os itens 1-8.
_____ (1) A leader is clearly recognized by all team members.	_____ (1) Um líder é claramente reconhecido por todos os membros da equipe.	___ (1) Um líder é claramente reconhecido por todos os membros da equipe.	_____ (1) O líder é claramente reconhecido por todos os membros da equipe.
_____ (2) The team leader assures maintenance of an appropriate balance between command authority and team member participation.	_____ (2) Um líder de equipe garante a manutenção de um equilíbrio apropriado entre comando autoritário e participação dos membros da equipe.	___ (2) O líder da equipe assegura a manutenção de um equilíbrio adequado entre a autoridade de comando e a participação dos membros da equipe.	_____(2) O líder de equipe garante a manutenção de um equilíbrio apropriado entre a autoridade de comando e a participação dos membros da equipe.
_____ (3) Each team member demonstrates a clear understanding of his or her role.	_____ (3) Cada membro da equipe demonstra um claro entendimento de suas atribuições (ou funções).	___ (3) Cada membro da equipe demonstra uma compreensão clara de seu papel.	_____ (3) Cada membro da equipe demonstra um claro entendimento de suas atribuições.
_____ (4) The team prompts each other to attend to all significant clinical indicators throughout the procedure/intervention.	_____ (4) A equipe orienta a cada um para atender a todos os indicadores clínicos significativos durante procedimentos/intervenções.	___ (4) A equipe orienta uns aos outros para atender a todos os indicadores clínicos significativos ao longo do procedimento/intervenção.	_____(4) A equipe orienta uns aos outros para atender a todos os indicadores clínicos significativos ao longo do procedimento/intervenção.
_____ (5) When team members are actively involved with the patient, they verbalize their activities aloud.	_____ (5) Quando os membros da equipe são envolvidos ativamente com o paciente, eles falam as suas atividades em voz alta.	___ (5) Quando os membros da equipe estão ativamente envolvidos com o paciente, eles verbalizam suas atividades em voz alta.	____ (5) Quando os membros da equipe estão ativamente envolvidos com o paciente, eles verbalizam suas atividades em voz alta.
_____ (6) Team members repeat back or paraphrase instructions and clarifications to indicate that they heard them correctly.	_____ (6) Os membros da equipe repetem ou parafraseiam instruções ou esclarecimentos para indicar o que eles ouviram corretamente.	___ (6) Os membros da equipe repetem ou parafraseiam instruções e esclarecimentos para indicar que eles ouviram corretamente.	_____(6) Os membros da equipe repetem ou parafraseiam instruções ou esclarecimentos para indicar que eles ouviram corretamente.
_____ (7) Team members refer to established protocols and checklists for the procedure/intervention.	_____ (7) Os membros de equipe indicam protocolos estabelecidos e checklists para o procedimento/intervenção.	___ (7) Os membros da equipe referem-se aos protocolos e listas de verificação estabelecidos para o procedimento/intervenção.	_____(7) Os membros de equipe indicam protocolos estabelecidos e checklists para o procedimento/intervenção.
_____ (8) All members of the team are appropriately involved and participate in the activity.	_____ (8) Todos os membros da equipe são devidamente envolvidos e participantes da atividade.	___ (8) Todos os membros da equipe estão adequadamente envolvidos e participar da atividade.	_____(8) Todos os membros da equipe estão devidamente envolvidos e participam da atividade.
Items 9-16 may be marked “NA (not applicable)” if no situations occurred in which these types of responses were required.	Os itens 9-16 podem ser marcados como “NA (não aplicável)” se não houver situações em que esses tipos de respostas sejam necessárias.	Os itens 9-16 podem ser marcados como “NA (não aplicável)” se não houver situações em que esses tipos de respostas sejam necessárias.	Itens 9-16 podem ser marcados como NA (não aplicável) se não houver situações em que esses tipos de respostas sejam necessárias.
_____ (9) Disagreements or conflicts among team members are addressed without a loss of situation awareness.	_____ (9) Desacordos ou conflitos entre os membros da equipe são abordados sem perda de controle da situação.	___ (9) Desentendimentos ou conflitos entre os membros da equipe são resolvidos sem perda de consciência da situação.	_____ (9) Desacordos ou conflitos entre os membros da equipe são resolvidos sem perda de controle da situação.
_____ (10) When appropriate, roles are shifted to address urgent or emergent events.	_____ (10) Quando apropriado, regras são mudadas para atender urgências ou eventos emergentes.	___ (10) Quando apropriado, os papéis são trocados para atender questões urgentes ou eventos emergentes.	___ (10) Quando apropriado, os papéis são trocados para atender questões urgentes ou eventos emergentes.
_____ (11) When directions are unclear, team members acknowledge their lack of understanding and ask for repetition and clarification.	_____ (11) Quando os comandos não são claros, os membros da equipe reconhecem sua falta de compreensão e pedem repetições ou esclarecimentos.	___ (11) Quando as instruções não são claras, os membros da equipe reconhecem sua falta de compreensão e pedem repetição e esclarecimento.	___ (11) Quando as instruções não são claras, os membros da equipe reconhecem sua falta de compreensão e pedem repetição e esclarecimento.
_____ (12) Team members acknowledge -in a positive manner-statements directed at avoiding or containing errors or seeking clarification.	_____ (12) Membros de equipe reconhecem - de maneira positiva -orientações destinadas a evitar ou conter erros, ou buscam esclarecimentos.	___ (12) Os membros da equipe reconhecem - de maneira positiva - declarações dirigidas a evitar ou conter erros ou buscar esclarecimentos.	_____ (12) Membros de equipe reconhecem - de maneira positiva - orientações destinadas a evitar ou conter erros, buscando esclarecimentos.
_____ (13) Team members call attention to actions that they feel could cause errors or complications.	_____ (13) Os membros da equipe prestam atenção nas ações que eles sentem que poderiam causar erros ou complicações.	___ (13) Os membros da equipe chamam a atenção para ações que percebem que podem causar erros ou complicações.	_____ (13) Os membros da equipe prestam atenção nas ações que eles sentem que poderiam causar erros ou complicações.
_____ (14) Team members respond to potential errors or complications with procedures that avoid the error or complication.	_____ (14) Os membros da equipe respondem por potenciais erros ou complicações com procedimentos que evitam erros ou complicações.	___ (14) Os membros da equipe respondem a possíveis erros ou complicações com procedimentos que evitam o erro ou complicação.	_____ (14) Os membros da equipe respondem por potenciais erros ou complicações com procedimentos para evitá-los.
_____ (15) When statements directed at avoiding or containing errors or complications do not elicit a response to avoid or contain the error, team members persist in seeking a response.	_____ (15) Quando declarações destinadas a evitar ou conter erros ou complicações não provocam uma resposta para evitar ou conter o erro, os membros da equipe persistem em encontrar uma resposta.	___ (15) Quando as afirmações para evitar ou conter erros ou complicações não provocam uma resposta para evitar ou conter o erro, os membros da equipe seguem buscando uma resposta.	. _____ (15) Quando as afirmações para evitar ou conter erros ou complicações não provocam respostas para evitar ou conter os erros, membros da equipe seguem buscando uma resposta.
_____ (16) Team members ask each other for assistance prior to or during periods of task overload.	_____ (16) Os membros da equipe pedem uns aos outros para se ajudarem mutuamente, antes ou durante períodos de sobrecarga de tarefas.	___ (16) Os membros da equipe pedem ajuda uns aos outros antes ou durante períodos de sobrecarga de tarefas.	_____ (16) Os membros da equipe pedem ajuda uns aos outros antes ou durante períodos de sobrecarga de tarefas.

Source: Prepared by the authors

The third stage was back-translation, which consists of translating the obtained versions back into the original language (English), to verify their validity and that the content is reflecting the same as the original versions. The agreement between the back-translation and the original version, in turn, does not guarantee a satisfactory direct translation, as there may be inaccuracies. Back-translation makes it possible to identify inconsistencies in the translation^17^. 

Two native English translators, without contact with the original tool and without contact between them (translators C and D), carried out this part, blinded to the original English version, working with the T1-2 version of the questionnaire, translating the document back into the English, creating the BT-1 and BT-2 versions^14^ ^-^ ^17^ (Table 2).

**Table 2:** BT1 and BT2 translations obtained from back translation.

ORIGINAL	BT1	BT2
TABLE 1. Mayo High Performance Teamwork Scale	TABLE 1. Mayo Work Scale on High Team Performance	Table 1. Mayo High Performance Teamwork Scale
Use the following scale to rate the team on each dimension:	Use the following scale to rate the team in each category:	Use the following scale to evaluate each dimension of yteam.
0 Never or Rarely	0 Never or Rarely	0 Never or Rarely
1 Inconsistently	1 Inconsistent	1 Inconsistently
2 Consistently	2 Consistent	2 Consistently
Please rate conservatively. Most teams that have not worked extensively together do not consistently demonstrate many of the qualities described in the scale.	Please rate carefully. Many teams that have not consistently worked together, fail to consistently exhibit many of the qualities described in this scale.	Please consider each point carefully. Many teams that have not worked together extensively fail to consistently show the qualities written below.
Always rate items 1-8	Always rate items 1-8.	Required: Items 1-8
_____ (1) A leader is clearly recognized by all team members.	_____ (1) There is a leader who is clearly recognized by all the members of team	_____ (1) A specific leader is clearly recognized by all members of the team.
_____ (2) The team leader assures maintenance of an appropriate balance between command authority and team member participation.	_____(2) The team leader maintains an appropriate balance between command authority and team participation.	_____(2) The team leader ensures that an appropriate balance is maintained between authoritative command and participation by team members..
_____ (3) Each team member demonstrates a clear understanding of his or her role.	_____ (3) Each team member demonstrates a clear understanding of their role.	_____ (3) Each team member demonstrates a clear understanding of their role (or roles).
_____ (4) The team prompts each other to attend to all significant clinical indicators throughout the procedure/intervention.	_____(4) team members prompt each other to attend to all clinically significant symptoms during procedures/interventions.	_____(4) The team advises each individual to comply with all significant clinical indicators during procedures/interventions.
_____ (5) When team members are actively involved with the patient, they verbalize their activities aloud.	____ (5) When team members are with a patient, they explain their actions to the patient out loud.	____ (5) When team members are actively involved with a patient, they verbalize their activities out loud.
_____ (6) Team members repeat back or paraphrase instructions and clarifications to indicate that they heard them correctly.	_____(6) team members repeat or paraphrase instructions to show that they understood them clearly.	_____(6) Team members repeat or paraphrase instructions or clarifications to indicate they heard correctly.
_____ (7) Team members refer to established protocols and checklists for the procedure/intervention.	_____(7) Team members refer to established protocols/checklists before starting a(an) procedure/intervention.	_____(7) Team members refer to established protocols and checklists for the procedure/intervention.
_____ (8) All members of the team are appropriately involved and participate in the activity.	_____(8) All of the team members are duly and properly involved in daily activities.	_____(8) All team members are properly involved and participate in the activity.
Items 9-16 may be marked “NA (not applicable)” if no situations occurred in which these types of responses were required.	Items 9-16 can be marked as “not applicable” if the situation(s) covered would not occur in your workplace.	Items 9-16 can be marked “NA (not applicable)” if there are no situations where these types of responses are required.
_____ (9) Disagreements or conflicts among team members are addressed without a loss of situation awareness.	_____ (9) Disagreements or conflicts between team members are resolved without losing control of the situation.	_____ (9) Disagreements or conflicts between team members are addressed without loss of control of the situation.
_____ (10) When appropriate, roles are shifted to address urgent or emergent events.	___ (10) When appropriate, team members' roles are switched to address pressing issues or unexpected events.	___ (10) When appropriate, roles are switched to address pressing issues or emerging events.
_____ (11) When directions are unclear, team members acknowledge their lack of understanding and ask for repetition and clarification.	___ (11) When instructions are not clear, team members recognize the need for more information and ask for the instructions to be repeated/clarified.	___ (11) When instructions are unclear, team members acknowledge their lack of understanding and ask for repetition and further clarification.
_____ (12) Team members acknowledge -in a positive manner-statements directed at avoiding or containing errors or seeking clarification.	_____ (12) Team members recognize - in a positive way - guidelines aimed at clarifying uncertainties or avoiding/preventing mistakes.	_____ (12) Team members recognize,in a positive way, guidelines aimed at avoiding or containing errors, or seeking clarification.
_____ (13) Team members call attention to actions that they feel could cause errors or complications.	_____ (13) Team members pay special attention to activities which (they feel) could lead to mistakes or complications.	______ (13) Team members pay attention to actions they feel could cause errors or complications.
_____ (14) Team members respond to potential errors or complications with procedures that avoid the error or complication.	_____ (14) Team members respond to potential mistakes/complications by using proper procedures.	_____ (14) Team members account for potential errors or complications with procedures that prevent errors or complications.
_____ (15) When statements directed at avoiding or containing errors or complications do not elicit a response to avoid or contain the error, team members persist in seeking a response.	_____ (15) When procedures that are intended to avoid or control mistakes/complications are not successful, team members persist in trying to find a solution.	_____ (15) When statements meant to avoid or contain errors or complications do not elicit a response to avoid or contain the error, team members persist upon finding an answer.
_____ (16) Team members ask each other for assistance prior to or during periods of task overload.	_____ (16) When overburdened, team members ask each other for help before and during their assignments.	_____ (16) Team members ask one another for help before or during periods of task overload.

Source: Prepared by the authors

The fourth stage, called the committee of experts, is crucial to achieve cross-cultural equivalence, as its role is to consolidate all versions of the questionnaire and develop the initial version of the questionnaire for field tests^17^. To validate the scale, as described in the methodology section, we invited experts in the study area, whose characterization is shown in Table 3.

**Table 3:** Characterization of the validator judges of the simulation scenarios.

Judge	Gender	Graduation	Time	Specialization	Time	Masters	Time	Doctorate	Time
1	Fem	Nursing	>10 years	Yes	> 10 years	Yes	6 to 10 years	Yes	6 to 10 years
2	Fem	Medicine	> 10 years	Yes	> 10 years	Yes	> 10 years	Yes	> 10 years
3	Fem	Medicine	6 to 10 years	Yes	2 to 5 years	Yes	< 2 years	No	-
Judge	Gender	Graduation	Time	Specialization	Time	Masters	Time	Doctorate	Time
4	Men	Medicine	> 10 years	Yes	> 10 years	No	-	No	-
5	Fem	Nursing	> 10 years	Yes	> 10 years	Yes	6 to 10 years	Yes	6 to 10 years
6	Men	Nursing	> 10 years	Yes	6 to 10 years	Yes	2 to 5 years	No	-
7	Men	Medicine	> 10 years	Yes	6 to 10 years	Yes	< 2 years	No	-
8	Fem	Medicine	> 10 years	Yes	6 to 10 years	Yes	2 to 5 years	No	-
9	Fem	Nursing	>10 years	Yes	> 10 years	Yes	>10 years	No	-
10	Men	Medicine	6 to 10 years	Yes	2 to 5 years	No	-	No	-
11	Fem	Nursing	>10 years	Yes	>10 years	Yes	>10 years	No	-
12	Fem	Medicine	>10 years	Yes	>10 AM Years	No	-	No	-
13	Fem	Medicine	6 to 10 years	Yes	2 to 5 years	No	-	No	-
14	Fem	Nursing	>10 years	Yes	6 to 10 years	Yes	6 to 10 years	Yes	2 to 5 years
15	Fem	Nursing	>10 years	Yes	>10 years	Yes	>10 years	Yes	>10 years
16	Fem	Physiotherapy	2 to 5 years	Yes	<2 years	Yes	2 to 5 years	Yes	2 to 5 years
17	Men	Medicine	>10 years	Yes	>10 years	Yes	>10 years	No	-
18	Men	Medicine	>10 years	Yes	2 to 5 years	Yes	2 to 5 years	Yes	2 to 5 years
19	Men	Medicine	>10 years	Yes	>10 years	No	-	No	-
20	Fem	Medicine	>10 years	Yes	2 to 5 years	Yes	>10 years	Yes	>10 years
21	Men	Medicine	2 to 5 years	Yes	2 to 5 years	Yes	2 to 5 years	No	-
22	Men	Nursing	>10 years	Yes	>10 years	Don't	-	No	-
23	Fem	Nutrition	>10 years	Yes	>10 years	Yes	2 to 5 years	No	-
24	Fem	Medicine	>10 years	Yes	2 to 5 years	No	-	Yes	2 to 5 years
25	Men	Medicine	>10 years	Yes	>10 years	No	-	Yes	2 to 5 years
26	Men	Medicine	>10 years	Yes	>10 years	Yes	6 to 10 years	No	-
27	Men	Medicine	6 to 10 years	Yes	2 to 5 years	Yes	2 to 5 years	No	-
28	Fem	Nursing	6 to 10 years	Yes	6 to 10 years	Yes	2 to 5 years	Yes	<2 years
29	Fem	Medicine	>10 years	Yes	2 to 5 years	Yes	2 to 5 years	Yes	<2 years
30	Fem	Nursing	6 to 10 years	Yes	2 to 5 years	Yes	2 to 5 years	Yes	<2 years
31	Men	Nursing	>10 years	Yes	2 to 5 years	Yes	2 to 5 years	No	-
32	Fem	Nursing	>10 years	Yes	>10 years	Yes	2 to 5 years	No	-
33	Fem	Medicine	>10 years	Yes	2 to 5 years	Don't	-	No	-
34	Men	Nursing	6 to 10 years	Yes	6 to 10 years	No	-	No	-
35	Fem	Medicine	>10 years	Yes	>10 years	Yes	2 to 5 years	No	-
36	Fem	Medicine	>10 years	Yes	6 to 10 years	Yes	6 to 10 years	No	-
37	Fem	Medicine	>10 years	Yes	>10 years	Yes	6 to 10 years	No	-
38	Men	Medicine	>10 years	Yes	>10 years	No	-	No	-
39	Men	Medicine	>10 years	Yes	2 to 5 years	Yes	6 to 10 years	Yes	<2 years
40	Fem	Physiotherapy	>10 years	Yes	>10 years	No	-	No	-

Source: Prepared by the authors.

The role of the expert committee is to review all versions of the questionnaire and to develop what would be considered the pre-final version of the questionnaire for Field Testing^17^. 

The committee of 40 experts reviewed all the translations, reaching a consensus that there was no discrepancy between the original version and the proposed version T1-2. The material available to the committee included the original scale and translation versions (Tables 1 and 2), along with the records of the corresponding discrepancies between the translations, marked in red, to facilitate the analysis and allow the proper appreciation of all stages of the process. The four reviewers and the observer also took part in this stage.

At this stage, each item of the scale was examined individually, so that specific attention was given to semantic, idiomatic, experiential, and conceptual equivalences, and for each of the equivalences, scores from 1 (strongly disagree) to 5 (strongly agree) were assigned on a Likert scale.

Although some inconsistencies were identified between BT-1, BT-2, and the original version, the expert committee examined each item of the scale, basing its assessments on the four versions of the scale T-1, T-2, BT-1, and BT-2. The discrepancies were considered irrelevant by the experts and a consensus was reached on the appropriate wording of each item, considering that the CVI for the equivalences analyzed was between 0.9 and 1.0, with no indications for modifications in the T1-2 version, which was thus admitted as a pre-final version. Checks were also made to ensure that the content was consistent with the CRM, and the experts found the content validity to be appropriate to the context in which the scale would be used.

Because it is a scale to evaluate teamwork in simulation-based education environments, and considering possible translation, sampling, and response biases, a correlation with interprofessional education was performed. Thus, the items of the scale were compared with the matrices of collaborative competencies of the United Kingdom and Canada, and we observed that the scale evaluates competencies of those matrices, namely, knowledge from practice, ethical practice, teamwork, interprofessional communication, clarity of roles, collaborative leadership, and conflict resolution, proving to be a powerful tool to assess teamwork and non-technical skills, demonstrating that it addresses interprofessional competencies^22^.

The fifth stage is the pre-test, which consists of the field test of the new questionnaire, seeking to use the preliminary version to obtain useful information about how the questionnaire items are interpreted and what is the applicability of the instrument. It is noteworthy that although this step provides some useful insight into the interpretation of the questionnaire items, it is not mandatory and is not intended to structure the validity, reliability, or response patterns^17^. 

To test the MHPTS scale in Portuguese, we created a sample consisting of eight medical students and four evaluators. These students had recently completed training in medical emergencies, so they could perform the simulated scenario based on their prior knowledge. All students and invited evaluators participated, and there were no inclusion/exclusion criteria. In addition, they filled out the scale by assisting a simulated scenario of care for polytrauma patients in an emergency unit.

We examined the distribution of responses to look for a high proportion of missing items or unique responses, offering useful insight into how the questionnaire items were interpreted^17^. Therefore, it was a matter of the research team’s verification of the definitive version, with 100% of the answers coinciding about teamwork during the scenario.

Regarding the additional tests for the retention of the psychometric properties of the questionnaire, we highlight that, according to Beaton^18^, they were not used since the scale was not designed from scratch, and the psychometric properties have already been tested and validated from the original scale in English. However, we admit that this may be a limitation of the study, as well as the small number of participants involved in this stage.

The last step was the submission and appraisal of all reports to the authors of the instrument, which consists of the validation of all documentation by the project developers and the final version of the translated scale (Table 4). It was a process audit, with all the necessary steps and reports followed. Although it is not appropriate at this time to change the content, the entire process was followed to obtain a reasonable translation.

**Table 4:** Final version of the translated scale Escala MHPTS validada para o Brasil (Mayo High Performance Teamwork Scale) Por favor, avalie cuidadosamente. Muitas equipes que não têm trabalhado extensivamente juntas não demonstraram, de forma consistente, muitas das qualidades descritas na escala. Sempre avalie os itens 1-8.

Escala MHPTS Parte I e II		Avaliação da performance da equipe em cada item durante as ações			
Escala MHPTS Parte I - Indicadores de avaliação		0 Nunca ou raramente	1 Inconsistente		2 Consistente
Assinale, de forma consistente e OBRIGATÓRIA, quando as muitas qualidades descritas em cada um dos 8 itens foram demonstradas nas ações					
1	O líder é claramente reconhecido por todos os membros da equipe.				
2	O líder garante a manutenção de um equilíbrio apropriado entre a autoridade de comando e a participação dos membros da equipe.				
3	Cada membro da equipe demonstra um claro entendimento de suas atribuições.				
4	A equipe orienta uns aos outros para atender a todos os indicadores clínicos significativos ao longo do procedimento/intervenção.				
5	Quando os membros da equipe estão ativamente envolvidos com o paciente, eles verbalizam suas atividades em voz alta.				
6	Os membros da equipe repetem ou parafraseiam instruções ou esclarecimentos para indicar que eles ouviram corretamente.				
7	Os membros da equipe indicam protocolos estabelecidos e checklists para o procedimento/intervenção.				
8	Todos os membros da equipe estão devidamente envolvidos e participam da atividade.				
Os itens 9-16 podem ser marcados como “NA (não aplicável)”, se não houver situações em que esses tipos de respostas sejam necessários.					
Escala MHPTS Parte II - Indicadores de avaliação		Avaliação da performance da equipe em cada item durante as ações			
Os itens abaixo podem ser marcados como “NA (não aplicável)”, se houver necessidade, de acordo com as situações demonstradas ou não pelas equipes		0 Nunca ou raramente	1 Inconsistente	2 Consistente	NA Não Aplicável
9	Desacordos ou conflitos entre os membros da equipe são resolvidos sem perda de controle da situação.				
10	Quando apropriado, os papéis são trocados para atender questões urgentes ou eventos emergentes.				
11	Quando as instruções não são claras, os membros da equipe reconhecem sua falta de compreensão e pedem repetição e esclarecimento.				
12	Os membros da equipe reconhecem - de maneira positiva - orientações destinadas a evitar ou conter erros, buscando esclarecimentos.				
13	Os membros da equipe prestam atenção nas ações que percebem que podem causar erros ou complicações.				
14	Os membros da equipe respondem por potenciais erros ou complicações e adotam procedimentos para evitá-los.				
15	Quando declarações para evitar ou conter erros ou complicações não provocam respostas que evitem ou contenham os erros, os membros da equipe seguem buscando uma resposta.				
16	Os membros da equipe pedem ajuda uns aos outros antes ou durante períodos de sobrecarga de tarefas.				

*source: Prepared by the authors adapted from Mayo High Performance Teamwork Scale.*

Finally, we should note that the complexity that permeates the adaptation of scales reinforces the argument that the translation of research instruments requires a delicate balance between fidelity to the original and relevance to the new context, a methodological challenge that goes beyond translation and touches on the essence of cross-cultural research^17^ ^,^ ^18^.

## CONCLUSION

The Brazilian Portuguese version of the MHPTS demonstrated adequate conceptual, semantic, and syntactic equivalence, revealing excellent potential for use in clinical simulation contexts. In addition, it proved to be easily and quickly applicable, and was considered valid by the committee of experts after following all the steps pertinent to translation and cultural adaptation.

The process of translating and cross-cultural adaptation of the scale included a series of carefully adhered to steps, as described by Beaton et al.^17^, so that the final result would be reliable and valuable to the technical-scientific community. 

Therefore, this contribution to the health care community, whose language is Brazilian Portuguese, remains through a valid, reliable instrument widely used in other languages.
